# Chlordecone exposure in women and time to pregnancy: the Timoun cohort study in Guadeloupe, French West Indies

**DOI:** 10.1186/s12940-025-01233-z

**Published:** 2025-10-16

**Authors:** Maryem Ben-Fares, Christine Monfort, Philippe Kadhel, Nathalie Costet, Florence Rouget, Léah Michineau, Jean-Pierre Thomé, Sylvaine Cordier, Luc Multigner, Charline Warembourg, Ronan Garlantézec

**Affiliations:** 1 Univ Rennes, Inserm, EHESP, Irset (Institut de recherche en santé, environnement et travail) – UMR_S 1085, Rennes, F-35000 France; 2 CHU de Guadeloupe, Univ Antilles, Inserm, EHESP, Irset (Institut de Recherche en Santé, Environnement et Travail) - UMR_S 1085, Pointe à Pitre, France; 3 Univ Rennes, CHU Rennes, EHESP, Irset (Institut de recherche en santé, environnement et travail) – UMR_S 1085, 9 Av. du Professeur Léon Bernard, Rennes, F-35000 France; 4 Center for Analytical Research and Technology (CART), Laboratory of Animal Ecology and Ecotoxicology (LEAE), Université de Liège, Liege, 4000 Belgium

**Keywords:** Chlordecone, Time to pregnancy, Fecundability, Discrete-time cox model, French west indies

## Abstract

**Background:**

Chlordecone is a persistent organochlorine insecticide that was widely used to control banana root borer in the French West Indies until 1993. Animal studies have reported an impact of chlordecone exposure on female fertility, but no data are available for humans. Here, we investigated the association between chlordecone exposure in women and time to pregnancy (TTP).

**Methods:**

We included 668 pregnant women from the Timoun mother-child cohort study performed in Guadeloupe between 2004 and 2007. TTP was measured with a questionnaire at the inclusion visit. Chlordecone concentrations in maternal blood samples were determined at the time of delivery. A discrete-time Cox model was used to estimate fecundability odds ratios (fOR) and their 95% confidence intervals (95% CIs), with adjustment for potential confounders. Sensitivity analyses were performed by modifying the study population and censoring criteria.

**Results:**

Chlordecone was detected in 91% of the study population, with a median concentration of 0.3 µg/L (IQR: 0.1–0.7). The third and fourth quartiles of chlordecone exposure were associated with significantly lower fecundability (fORa [95% CI] = 0.76 [0.58, 0.99]; fORa [95% CI] = 0.72 [0.55, 0.95], respectively). A significant dose-dependent relationship was observed between chlordecone exposure and TTP (*p*-trend = 0.01). Similar results were observed in all sensitivity analyses except that for primiparous women.

**Conclusion:**

Our study supports the hypothesis that chlordecone affects the fertility of women and is therefore a public health concern in widely contaminated areas, such as the French West Indies.

**Supplementary Information:**

The online version contains supplementary material available at 10.1186/s12940-025-01233-z.

## Highlights

• Chlordecone is an organochlorine pesticide used in the French West Indies until 1993.

• Chlordecone affects female fertility in animals, but this has not yet been studied in humans.

• Chlordecone was detected in 91% of blood samples from pregnant women in the Timoun cohort.

• Chlordecone exposure is associated with a dose-response trend towards longer time to pregnancy in women.

**Supplementary Information:**

The online version contains supplementary material available at 10.1186/s12940-025-01233-z.

## Introduction

The French West Indies is one of the few areas in the world in which chlordecone, an organochlorine pesticide, was widely used, until 1993, to control banana root borer. This pesticide undergoes no significant biotic or abiotic degradation in the environment, resulting in permanent soil and water pollution. As a result, humans continue to be exposed to this chemical, mostly through the ingestion of contaminated foods, the primary source of exposure [1–3]. Several studies since 1999 have reported the detection of chlordecone in the blood of 90% of the general population of the French West Indies [4–6].

The half-life of chlordecone in human blood has been estimated at 96 to 165 days, with a mean value of 131 days [7, 8]. Chlordecone preferentially accumulates in the liver, unlike most other POPs, which accumulate principally in fatty tissues [8]. Chlordecone is a recognized endocrine-disrupting chemical (EDC) with estrogenic and progestogenic hormone-like properties clearly demonstrated both in vitro and in vivo [9, 10]. Experimental studies in animals have shown that chlordecone is reprotoxic, fetotoxic, carcinogenic and neurotoxic [11–14]. Rodent studies have consistently reported that chlordecone exposure impairs ovulation and decreases the size of the ovarian reserve, with an increase in the number of atretic antral follicles and a decrease in the number of normal follicles of median size, thereby affecting ovum viability, and that it alters implantation, resulting in an overall decrease in fertility [15–19].

In humans, fertility is defined as the biological capacity of a man, woman, or couple to conceive a child. Time to pregnancy (TTP) is an indicator of fertility widely used in epidemiology. TTP is defined as the period between the cessation of contraception and the clinical confirmation of a pregnancy in a couple planning a pregnancy and engaging in regular sexual intercourse. TTP is used to estimate fecundability, defined as the probability of conception in a given month or menstrual cycle [20–22].

Animal studies have revealed adverse effects of chlordecone exposure on female fertility, but no such epidemiological studies have been performed in humans to determine whether similar effects occur in women. We therefore investigated the association between chlordecone exposure in women and TTP.

## Methods

### Study population

We used data from the Timoun mother-child cohort. In total, 1,071 pregnant women from the general population of Guadeloupe in the French West Indies were enrolled in the cohort at second or third-trimester check-up visits between November 2004 and December 2007 at public health centers (Pointe-à-Pitre University Hospital, Basse-Terre General Hospital, and antenatal care units). Women were eligible for inclusion in the cohort if they had lived in Guadeloupe for at least three years and planned to give birth at the maternity hospital in Pointe-à-Pitre or the maternity hospital in Basse-Terre. Women with a history of epilepsy or long-term corticosteroid treatment at the time of inclusion were excluded from the cohort. Data were collected with a questionnaire administered by midwives at the inclusion visit. This questionnaire recorded sociodemographic characteristics, including maternal age, education, and country of birth. Women were also asked about their contraceptive history, any fertility treatments used, their occupation, obstetric and medical history, their use of tobacco, drugs and alcohol and other lifestyle habits. At delivery, a maternal blood sample was collected for chlordecone determination. The study was approved by the Guadeloupean Ethics Committee for studies involving human subjects (Project no. 03–04 01/10/2004). Each participant provided written informed consent.

### Time to pregnancy

TTP was determined retrospectively at the time of inclusion. Participants were asked to report how long, in months, it took them to conceive for the pregnancy underway. TTP was recorded as a discrete variable, the number of months of regular, unprotected sexual intercourse between stopping contraception and conception. Pregnancies that occurred immediately after stopping contraception were assigned a TTP of one month.

We restricted the TTP analysis to women with planned pregnancies. Planned pregnancies were identified on the basis of the woman’s responses to questions about contraceptive use, type of contraception, whether or not the method had been stopped and the date on which it was stopped if this was done voluntarily. Based on this information, we excluded 139 women whose pregnancies were unplanned and 84 women for whom pregnancy planning status could not be determined. We studied a total of 843 women with planned pregnancies and available TTP data (*n* = 5 missing TTP) (see Appendix Fig. 2).

### Chlordecone exposure

Chlordecone concentrations were measured in 10 mL samples of maternal blood collected in EDTA-coated tubes at delivery. The blood samples were centrifuged and the resulting plasma samples were stored at −30 °C until shipment to the Analytical and Technological Research Center in Liège, Belgium. Chlordecone levels were determined by high-resolution gas chromatography coupled with electron capture detection, with a limit of detection (LOD) of 0.02 µg/L. Details of sampling, analyses, and quality assurance and control are provided elsewhere [5]. We selected only those women with planned pregnancies for whom chlordecone determinations were available (*N* = 668), corresponding to 62% of the Timoun cohort population (see Appendaix Fig. 2).

### Statistical analysis

#### Main analysis

Descriptive statistics were calculated for participant characteristics, chlordecone concentrations and TTP. Levels of chlordecone below the limit of detection (LOD) (~ 9%) were imputed with a simple maximum likelihood imputation method [23].

We investigated the association between chlordecone exposure and TTP, using a discrete-time Cox proportional hazards model to estimate fecundability odds ratios (fOR). This model compares the odds of conceiving in a given month for the exposed group to the odds of conceiving in a given month for the non-exposed group, conditional on not having conceived in the previous month. A fOR below 1 indicates a longer TTP and lower fecundability (i.e. a lower probability of conception in a given month). As recommended [24], we censored the TTP data at 13 months when TTP exceeded 12 months (19% of women, *n* = 129) and at the self-reported TTP when couples sought medical assistance due to difficulties conceiving (< 1% of women, *n* = 6), considering this TTP to be the minimal value (as if they had not used medical assistance).

A causal directed acyclic graph (DAG) was used to identify potential confounders (see Appendix Fig. 3). Based on the DAG, the following variables were selected: maternal age, body mass index (BMI), type of contraception, and smoking status. These variables were calculated for the start of attempt to get pregnant, as follows: maternal age was determined by subtracting the date of delivery from the date of contraceptive cessation, BMI was calculated from the women’s self-reported weight and height before pregnancy, and maternal smoking was assessed based on information about smoking habits, the date of smoking cessation and the date of contraception cessation reported by the women. Missing data on covariates (*n* = 7 for BMI, *n* = 4 for contraceptive use, and *n* = 6 for smoking habits) were replaced with the mode of the corresponding variable.

The assumptions of the Cox model were verified. The log-linearity of continuous variables was assessed with Martingale residuals, which indicated that the associations between TTP and chlordecone, age, and BMI were not log-linear. As a result, chlordecone exposure was modeled as a categorical variable based on quartiles, whereas age and BMI were each categorized into three classes: maternal age (< 25 years; 25–35 years; >35 years) and BMI (< 25 kg/m²; 25–30 kg/m²; ≥30 kg/m²). The proportional hazards assumption was evaluated with Schoenfeld residuals; the assumption held for all variables except for type of contraception. A stratified Cox model was therefore used to account for heterogeneity in the baseline hazard function according to the type of contraception used before trying to conceive (oral contraception vs. other methods).

We assessed the dose-dependent relationship between chlordecone exposure in quartiles and TTP, with tests for a linear trend across chlordecone exposure categories, with this exposure in quartiles treated as a continuous variable in the Cox model.

#### Sensitivity analysis

We performed a series of sensitivity analyses to assess potential bias, as recommended in TTP studies [24, 25]. Models were tested by: (1) restricting the analysis to primiparous women, (2) excluding women who conceived in the first month after the cessation of contraception (TTP = 1), (3) excluding women using fertility treatments, and (4) censoring TTP at 7, 10, and 15 months instead of 13 months. An additional analysis was then performed with adjustment for the other covariates associated with TTP (origin, education and marital status). The results of these analyses were compared with our main analysis, to assess its robustness.

R software version 4.3.0 was used for all statistical analyses.

## Results

The characteristics of the study population are presented in Table 1. The median age of the 668 women included in our analysis at the start of the attempt to conceive was 30 years (IQR: 24.6–34.4 years) and 80% of these women were born in Guadeloupe or Martinique. Half the women were educated to at least baccalaureate (high-school diploma) level (49%), did not live with their partner (45%), used the pill as a contraceptive method (51%), and more than one third were primiparous (36%). Very few of the women reported the use of tobacco (9%), drugs (1%) or alcohol (2%).

**Table 1:** Characteristics of the study participants (*N*=668), from the Timoun study, Guadeloupe, French West Indies, 2004-2007

Variables	*N*	Percentage	Median (IQR)
Maternal age at pregnancy attempt (years)			30.3 (24.6–34.4)
< 25	180	27%	
25–35	336	50%	
>35	152	23%	
Origin			
French West Indies	533	79.8%	
Other Caribbean islands	56	8.4%	
Europe	79	11.8%	
Education			
Primary or none	30	4.5%	
Secondary	309	46.3%	
High-school diploma and higher	329	49.2%	
Marital status			
Living with partner	359	55.1%	
Living alone	164	25.1%	
Living with own family	129	19.8%	
Missing data	16		
Prepregnancy BMI (kg/m ^2^)			23.8 (21.2–28.1)
< 25	391	59%	
25–30	146	22%	
≥ 30	124	19%	
Missing data	7		
Type of contraception used before pregnancy			
Oral contraception	338	51%	
Other methods	326	49%	
Missing data	4		
Parity			
0	243	36%	
1	211	32%	
2 or more	214	32%	
Fertility treatment			
No	650	97.2%	
Yes	18	2.8%	
Missing data	27		
Smoker of cigarettes at time of pregnancy attempt			
No	604	91.2%	
Yes	58	8.8%	
Missing data	6		
Alcohol consumption during pregnancy			
No	631	97.7%	
Yes	15	2.3%	
Missing data	22		
Drug use at time of pregnancy attempt			
No	661	99%	
Yes	7	1.0%	
Plasma chlordecone concentration (µg/L)	668	100%	0.3 (0.1–0.7)
Above LOD (*≥* 0.02 µg/L)	605	90.5%	
TTP (months)	668	100%	4 (2–12)
>12 months	129	19%	

*Abbreviations:BMI *body mass index, *TTP *time to pregnancy, *SD *standard deviation, *IQR* interquartile range, *LOD* limit of detection

Chlordecone was detected in 91% of the study population, at a median concentration of 0.3 µg/L (IQR: 0.1–0.7). The median TTP was 4 months (IQR: 2–12 months) and the rate of infertility, defined as a TTP greater than 12 months, was 19%.

In this study population, higher education and marital status (living with the woman’s own family) were associated with a shorter TTP whereas a higher BMI was associated with a longer TTP (see Appendix Table 3).

The results of the analysis of the association between chlordecone exposure and TTP are presented in Table 2. We observed a statistically significant association for the third (0.38 and 0.81 µg/L) and fourth (≥ 0.81 µg/L) quartiles of chlordecone concentration (fORa [95% CI] = 0.76 [0.58, 0.99]; fORa [95% CI] = 0.72 [0.55, 0.95]), corresponding to decreases in fecundability of 24% and 28%, respectively, relative to the first quartile with a concentration no greater than 0.17 µg/L. In addition, a statistically significant dose-dependent inverse relationship was observed between chlordecone levels and fecundability (*p*-trend = 0.01).

**Table 2:** Crude and adjusted fOR for association between maternal blood Chlordecone concentration at delivery and TTP

		Crude			Adjusted^a^		
Chlordecone levels, µg/L	*N*	**fOR**	**95% CI**	*p*-value	**fOR**^b^	**95% CI**^b^	*p*-value
≤ 0.17	171	1			1		
0.17–0.38	163	0.89	0.68–1.16	0.40	0.88	0.67–1.15	0.36
0.38–0.81	176	0.75	0.58–0.98	0.03	0.76	0.58–0.99	0.04
≥ 0.81	158	0.74	0.56–0.96	0.02	0.72	0.55–0.95	0.02
*p*-trend			0.01		0.01		

^a^ Fecundability odds ratio adjusted for age at the start of the attempt to conceive, pre-pregnancy BMI and cigarette smoking at the start of the attempt to conceive, stratified by type of contraception

^b^ fOR and its 95% CIs estimated with a discrete-time Cox proportional hazards model with simple imputation (by mode) for missing covariate data

**Fig. 1:**
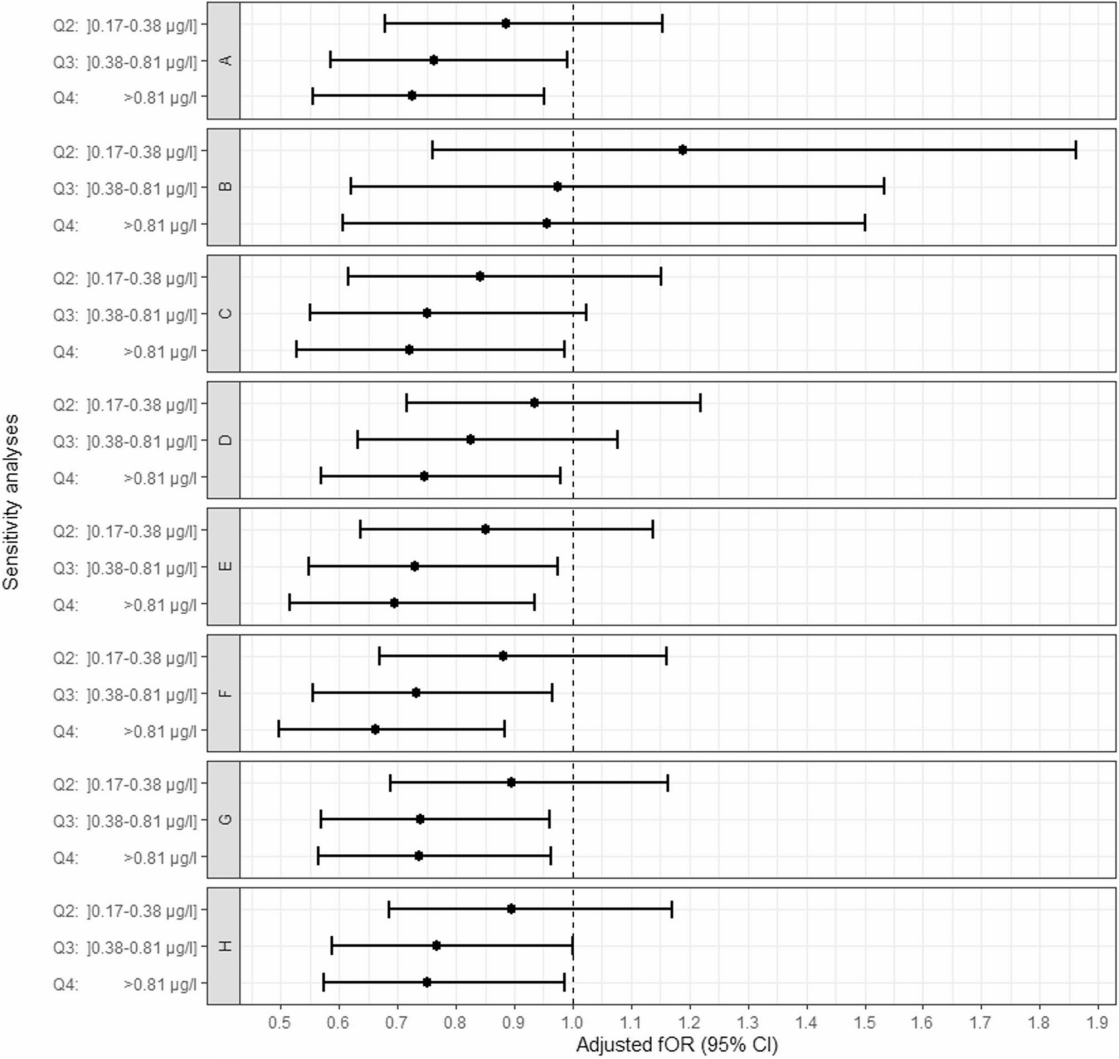
Sensitivity analysis. Points represent adjusted fOR and lines represent their 95% CIs for the association between chlordecone concentration and TTP in various sensitivity analyses. Models: **A)** main analysis (*n*=668);**B**) analysis restricted to primiparous women (*n*=243);**C**) analysis excluding women who conceived in the first month after the cessation of contraception with TTP=1 (*n*=510) ; **D**) analysis excluding women using fertility treatment (*n*=650); Main analysis with censoring at: **E**) 7 months, **F**) 10 months and **G**) 15 months (*n=*668) ; H) adjustment for covariate determinants of TTP (origin, education and marital status) (*n*=668)

**Fig. 2:**
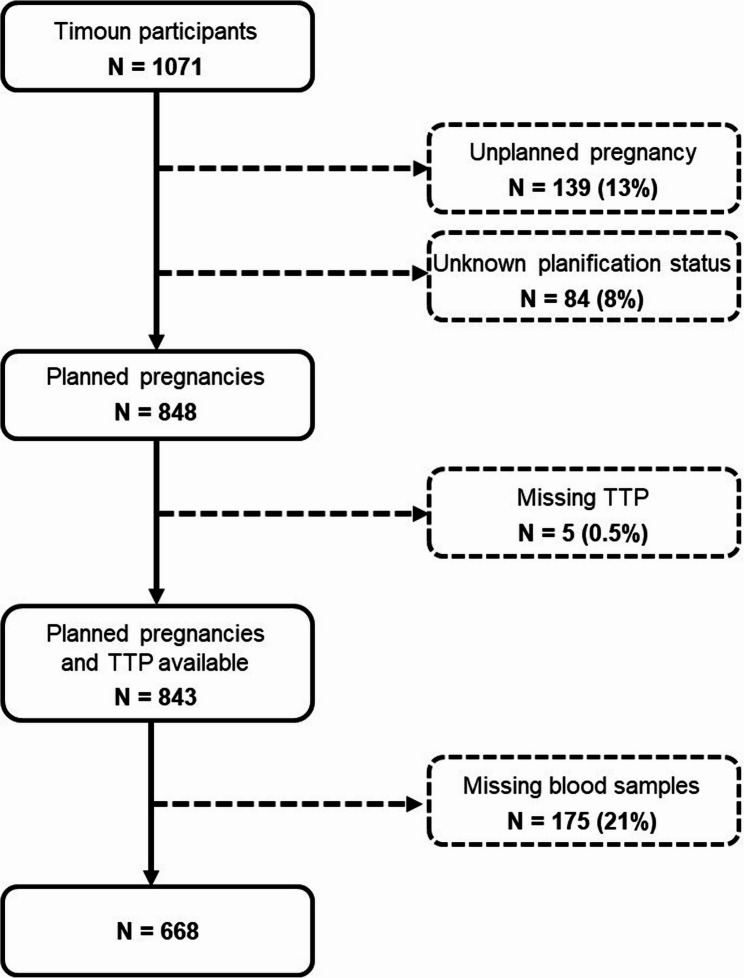
Flowchart of the study sample. Timoun mother-child cohort study, Guadeloupe, French West Indies, 2004-2007

Similar results were obtained in all sensitivity analyses (Fig. 1) except that restricted to primiparous women (Fig. 1, B). The overall effect of chlordecone on TTP disappeared in this analysis, due to a loss of the dose-dependent relationship for the second quartile of chlordecone exposure relative to the first quartile, but this relationship persisted between the second, third and fourth quartiles. The characteristics of the primiparous women are detailed in Appendix Table 4. Primiparous women were significantly younger, had higher education levels, were less likely to be from the Caribbean Islands, less likely to live with a partner, and more likely to have undergone fertility treatments than multiparous women. Other characteristics, including chlordecone levels, did not differ significantly between the two groups

.

## Discussion

This is the first study to investigate the relationship between chlordecone exposure and TTP in women. We found that chlordecone exposure in pregnant women was associated with a longer TTP, with a significant dose-dependence. This finding remained stable in all the sensitivity analyses performed except that limited to primiparous women.

Our findings are consistent with those of experimental studies in animals, which have reported harmful effects of chlordecone on female fertility. One study reported an association between chlordecone exposure and deleterious effects on gametes and embryos in female rabbits [26]. Another reported that chlordecone can cause ovarian tissue atrophy in female quails [27]. Several studies in rodents have shown that exposure to chlordecone induces early vaginal opening, prolonged vaginal estrus and anovulation [17, 19, 28–30]. These studies also reported that chlordecone caused the atresia of large antral follicles, a decrease in the number of healthy medium-sized and large follicles and an increase in the percentage of antral follicles. These ovulatory and postovulatory effects have been attributed to the well-known estrogenic properties of chlordecone [29]. For example, chlordecone inhibits the proestrus release of luteinizing hormone (LH) in ovariectomized mice [16] and causes the translocation of estrogen receptor sites to the uterine nucleus; it also increases uterine weight and stimulates the synthesis of the progesterone receptor, an estrogen receptor-mediated process, in immature rats. Chlordecone has been shown to be an agonist of estrogen receptor-α (ERα) [31], which is strongly expressed in antral follicles [32] potentially accounting for the increase in follicular atresia associated with chlordecone exposure [33].

Our results are also consistent with those of other studies in women exposed to other persistent organic pollutants (POPs), some of which have estrogenic properties. Several studies have used retrospective TTP data. A dose-dependent decrease in fecundability and an increase in the risk of infertility associated with serum dioxin levels were found in the Seveso Women’s Health Study (SWHS) (Italy, 1996-1998) [34] and a decrease in fecundability in women exposed to polychlorinated biphenyls (PCB138, PCB118, and PCB170) was observed in women from the PELAGIE cohort (France, 2002-2006)) [35]. Other studies using prospective TTP data have shown a significant decrease in fecundability associated with exposure to certain PCBs (PCB118, PCB167, and PCB209) in women from the LIFE study (USA, 2005-2007) [36], and associations, albeit not statistically significant, between the total concentration of all PCB congeners and low fecundability in the New York Angler Cohort Study (USA, 1991-1994) [37].

This study has several limitations, the first being that the study population included only women whose pregnancies resulted in a live birth. Thus, women experiencing difficulties conceiving and infertile women were underrepresented. This may limit the generalizability of our results and may have biased our results toward the null hypothesis [38]. The retrospective reporting of TTP in our study may be a source of recall bias. This bias is likely to be non-differential as the women did not know their blood chlordecone concentrations. Moreover, such a bias, if it indeed occurred, would be minimal because women reported TTP for the index pregnancy, and previous studies have reported a high validity of TTP recall even 10 to 15 years after the pregnancy [39–41]. There may also have been a digit-preference bias (i.e. a tendency to report a TTP value rounded up or down to values such as 3, 6, 12 or 18 months) [42, 43]. Nevertheless, the observed TTP distribution suggests that this bias was probably minimal, with no overrepresentation of these rounded values in the distribution, except for the longest TTP, for which censoring at 13 months limits the effect. Furthermore, analyses with different censoring dates (at 7, 10, and 15 months) had no impact on the results. Finally, we were unable to ensure that women did not include miscarriages (whether or not clinically recognized) in their self-reported TTP.

 TTP cannot be calculated for women with unplanned pregnancies, justifying the exclusion of unplanned pregnancies from this study [41, 42]. The frequency of unplanned pregnancies in the Timoun study (13%) was similar to that in similar studies by Chevrier et al. (12%) based on the same questionnaire[35],and by Vélez et al. (11%) [44]. However, it is more than double the 6% rate reported by Premranjith et al. [45]. Women with unplanned pregnancies are generally more fertile. The second sensitivity analysis assessed this pregnancy planning bias by excluding women who conceived in the first month after stopping contraception (TTP=1), suggesting an absence of impact in this study.

TTP measures the fertility of the couple and may provide information about the final common pathway for many biological events in women (e.g., ovarian reserve, dysovulation) and men (e.g., sperm quality). It may be influenced by factors present in either partner. A study in the male population of Guadeloupe found no significant association between blood chlordecone concentration and sperm parameters [46], so the absence of consideration of chlordecone concentrations in the blood of the fathers in our study is unlikely to be a major factor affecting the results. Nevertheless, other paternal factors, such as age and BMI, may still affect TTP. Furthermore, we adjusted for confounding factors selected *a priori* (age, BMI, smoking status and type of contraception) but we were unable to adjust for some potential determinants of TTP, such as alcohol or drug use, in our primary model. However, the very small proportion of women consuming these substances suggests that these factors would be unlikely to affect the results. Finally, we conducted a sensitivity analysis by adjusting for other TTP determinants — specifically origin, education and marital status — and the results were unaffected.

The principal confounding factors were included in our DAG analysis, but we were unable to exclude residual confounding, relating to co-exposure to other chemicals in particular. However, the levels of other chemicals known to be associated with an increase in TTP, such as other POPS or heavy metals, were found to be very poorly correlated with chlordecone levels in our study (Spearman’s Rho ≤0.2) (data not shown).

All but one of the sensitivity analyses confirmed the results of the main analysis. The exception, the sensitivity analysis restricted to primiparous women, yielded imprecise results that were unexpected given the findings of the main analysis. The second quartile of chlordecone concentration was associated with a non-significant increase in fecundability relative to the first quartile (reference), whereas the dose-dependent relationship according to which fecundability decreased with increasing exposure remained consistent in the second, third and the fourth quartiles. We compared the characteristics of the women in the first quartile with those of the women in the other quartiles and between primiparous and multiparous women, but we found no clear differences that might explain these differences in estimates. The sample size for this study was relatively small, corresponding to only one third of the main analysis population. Furthermore, in the analysis restricted to primiparous women, other adjustment variables, such as advanced age, higher BMI and tobacco use, appeared to be associated with higher fecundability, highlighting the specificity of this population. Some researchers have recommended limiting such analyses to primiparous women, but their utility remains a matter of debate [47, 48]. Moreover, this approach seems particularly relevant for prospective study designs and for studies of lipophilic pollutants stored in fatfor which changes in concentrations related to body weight changes, pregnancy and breastfeeding are known to occur. However, chlordecone is not stored in significant amounts in fat [49]. The contradictory results obtained in the analysis limited to primiparous women are therefore more likely to be due to the small sample size, consistent with the absence of associations with the other determinants of TTP. The determination of maternal blood chlordecone concentration, which accounts for all potential routes of exposure, is considered the gold standard method for evaluating exposure as it is much more reliable than indirect procedures based on dietary questionnaires, which may be subject to recall bias. We measured exposure at delivery rather than at conception or during pregnancy. However, given the half-life of chlordecone in human blood (between 96 and 165 days) [7, 8] and the absence of any policy to reduce exposure at the time of the study, a single determination of blood chlordecone concentration can be considered to reflect the body burden under steady-state conditions, thereby providing a sufficiently confident estimation of exposure over an extended period. However, we cannot exclude the possibility of measurement error for chlordecone exposure due to physiological changes associated with pregnancy.

## Conclusion

 Chlordecone exposure in pregnant women was found to be associated with lower fecundability, as indicated by a longer TTP. Our findings support the hypothesis that chlordecone affects the fertility of women. Additional studies are needed to confirm these results. Such studies could include the prospective follow-up of couples planning a pregnancy or investigations of the potential underlying causes of female infertility, such as a diminished ovarian reserve, as suggested by toxicological studies on chlordecone. If the results of this study are confirmed, public policies for reducing chlordecone exposure levels should be strengthened, particularly for women of childbearing age.

## Supplementary Information

Supplementary Material 1.

Supplementary Material 2.

Supplementary Material 3.

Supplementary Material 4.

## Figures and Tables

**Fig. 3:**
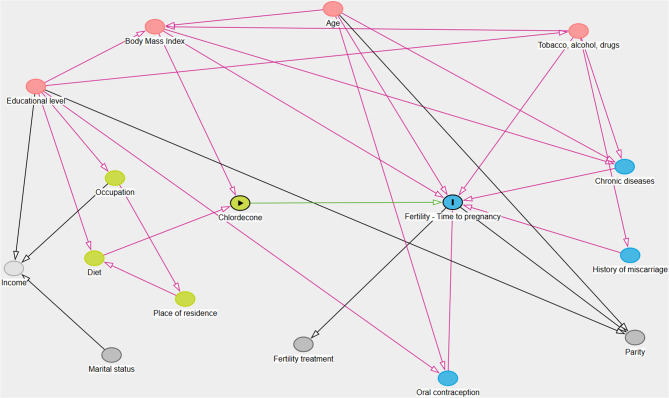
DAG chlordecone and time to pregnancy

## Data Availability

We cannot provide the research data as they include sensitive individual data, which are protected by strict national and European confidentiality rules. As such, the data cannot be shared or published.

## Appendix

**Table 3:** Participant characteristics and association with TTP

	Time to pregnancy (months)				
	*N*(%)		**Median (IQR)**	**Crude fOR** ^a^ **(95% CI)**	*p*-value
Total	668 (100%)		4 (2–12)		
Maternal age at pregnancy attempt (years)					
< 25	180 (27%)		4 (1–12)	1.00	
25–35	336 (50%)		4 (2–12)	0.89 (0.71–1.12)	0.33
>35	152 (23%)		4 (2–12)	0.93 (0.71–1.21)	0.62
Origin					
French West Indies	533 (79.8%)		4 (2–12)	1.00	
Other Caribbean Islands	56 (8.4%)		6 (2–12.2)	0.81 (0.57–1.14)	0.24
Europe	79 (11.8%)		3 (1.5–10.5)	1.10 (0.81–1.48)	0.51
Education					
Primary or none	30 (4.5%)		8 (2.2–12.7)	1.00	
Secondary	309 (46.3%)		4 (2–12)	1.32 (0.82–2.12)	0.24
High-school diploma and higher	329 (49.2%)		4 (2–12)	1.44 (0.89–2.31)	0.12
Marital status					
Living with partner	359 (55.1%)		5 (2–12)	1.00	
Living alone	164 (25.1%)		4 (1–12)	1.19 (0.94–1.49)	0.13
Living with own family	129 (19.8%)		2 (1–8)	1.49 (1.16–1.91)	0.001
Missing data	16				
Prepregnancy BMI (kg/m ^2^)					
< 25	391 (59%)		4 (2–12)	1.00	
25–30	146 (22%)		4 (2–12)	1.00 (0.79–1.26)	0.98
≥ 30	124 (19%)		6 (2–13)	0.74 (0.57–0.96)	0.02
Missing data	7				
Type of contraception used before pregnancy					
Oral contraception	338 (51%)		4 (2–12)	^b^	^b^
Other methods	326 (49%)		4 (1–12)	^b^	^b^
Missing data	4				
Parity					
0	243 (36%)		4 (1–12)	1.00	
1	211 (32%)		4 (2–12)	1.05 (0.84–1.32)	0.61
2 or more	214 (32%)		4 (2–12)	0.90 (0.71–1.13)	0.36
Smoker of cigarettes at time of pregnancy attempt					
No	604 (91.2%)		4 (2–12)	1.00	
Yes	58 (8.8%)		5.5 (2–12)	0.83 (0.59, 1.17)	0.29
Missing data	6				
Alcohol consumption during pregnancy					
No	631 (97.7%)		^c^	^c^	^c^
Yes	15 (2.3%)		^c^	^c^	^c^
Missing data	22				
Drug use at time of pregnancy attempt					
No	661 (99%)	^c^		^c^	^c^
Yes	7 (1.0%)	^c^		^c^	^c^

*Abbreviations:**fOR *fecundability odds ratio, *SD *standard deviation, *CI *confidence interval, *BMI *body mass index, *IQR *interquartile range

^a^ crude fOR and its 95% CI estimated with a Cox proportional hazards model for discrete time

^b^ no fOR is given for the type of contraception variable, as the proportional hazards assumption was not satisfied for this variable

^c^ no data are provided for drug and alcohol use, as the number of women consuming these substances was very small

**Table 4:** Comparative analysis of the general characteristics of primiparous and multiparous women

	Multiparous (*N* = 425)	Primiparous (*N* = 243)	*p*-value ^a^
Maternal age at pregnancy attempt (years)			< 0.001
< 25	53 (12.5%)	127 (52.3%)	
25–35	237 (55.8%)	99 (40.7%)	
>35	135 (31.8%)	17 (7.0%)	
Origin			0.05
French West Indies	342 (80.5%)	191 (78.6%)	
Other Caribbean Islands	41 (9.6%)	15 (6.2%)	
Europe	42 (9.9%)	37 (15.2%)	
Education			< 0.001
Primary or none	24 (5.6%)	6 (2.5%)	
Secondary	228 (53.6%)	81 (33.3%)	
High-school diploma and higher	173 (40.7%)	156 (64.2%)	
Marital status			< 0.001
Living with partner	256 (62.0%)	103 (43.1%)	
Living alone	117 (28.3%)	47 (19.7%)	
Living with own family	40 (9.7%)	89 (37.2%)	
Missing data	12	4	
Prepregnancy BMI (kg/m ^2^)			0.06
Mean (SD)	25.5 (5.7)	24.6 (6.0)	
Missing data	6	1	
Type of contraception used before pregnancy			0.96
Other methods	208 (49.2%)	118 (49.0%)	
Oral contraception	215 (50.8%)	123 (51.0%)	
Missing data	2	2	
Fertility treatment			0.03
No	400 (98.3%)	223 (95.3%)	
Yes	7 (1.7%)	11 (4.7%)	
Missing data	18	9	
Smoker of cigarettes at time of pregnancy attempt			0.57
No	387 (91.7%)	217 (90.4%)	
Yes	35 (8.3%)	23 (9.6%)	
Missing data	3	3	
Alcohol consumption during pregnancy			0.40
No	403 (98.1%)	228 (97.0%)	
Yes	8 (1.9%)	7 (3.0%)	
Missing data	14	8	
Drug use at time of pregnancy attempt			0.25
No	422 (99.3%)	239 (98.4%)	
Yes	3 (0.7%)	4 (1.6%)	
Plasma chlordecone concentration (µg/L)			0.29
Median (IQR)	0.39 (0.17–0.81)	0.38 (0.16–0.71)	
TTP (months)			0.61
Median (IQR)	4.0 (2.0–12.0)	4.0 (1.0–12.0)	

^a^ Student’s *t*-test or Mann-Whitney *U* test for quantitative variables, and Chi-squared test or Fisher’s exact test for qualitative variables
