# Supplementary figures and images for: Chlordecone exposure in women and time to pregnancy: the Timoun cohort study in Guadeloupe, French West Indies
Source: Environ Health. 2025 Oct 16;24:78. doi: 10.1186/s12940-025-01233-z (PMC12529803; doi:10.1186/s12940-025-01233-z)

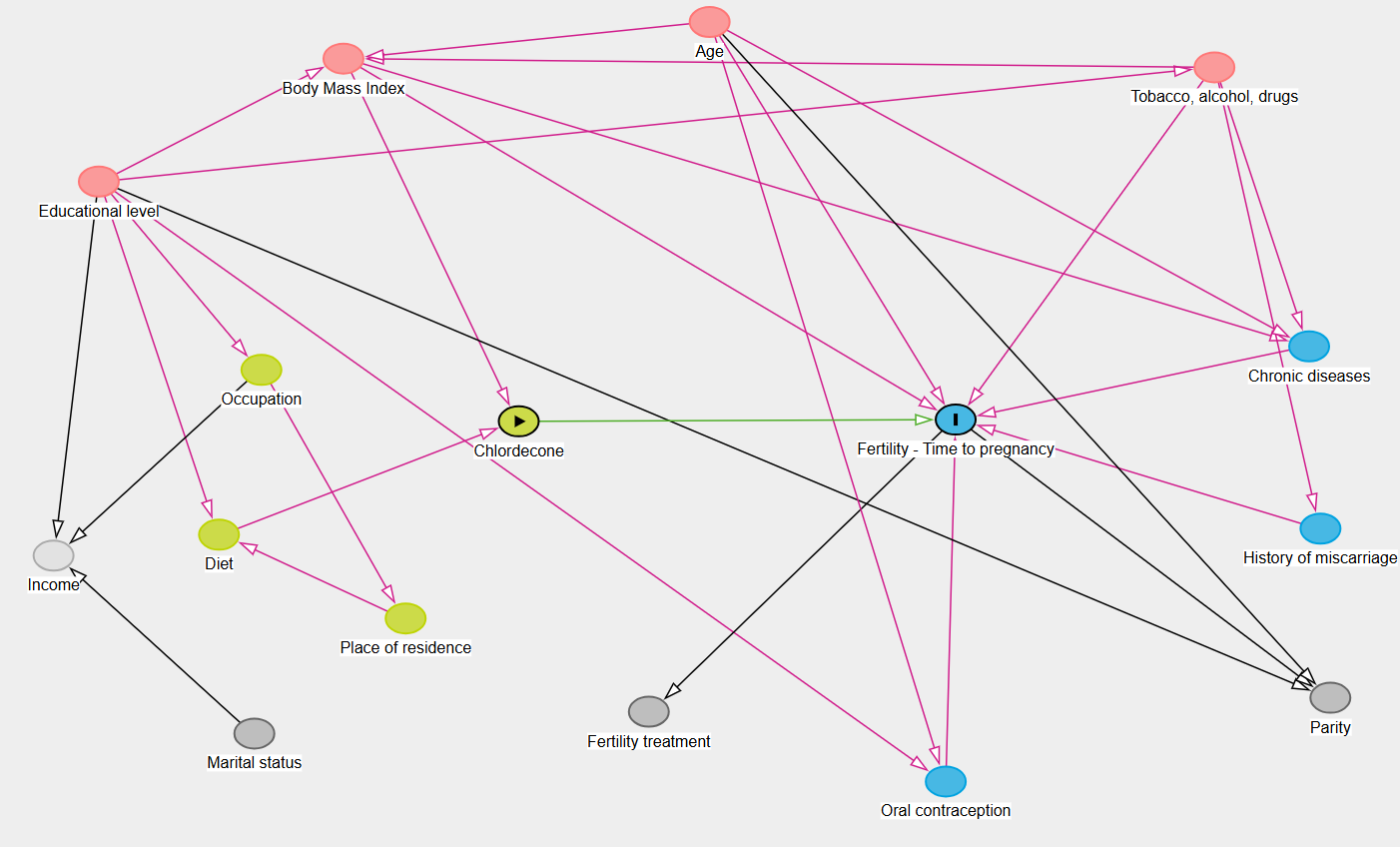

Supplement: Supplementary file 1 — Supplementary Material 1. [file 12940_2025_1233_MOESM1_ESM.png]

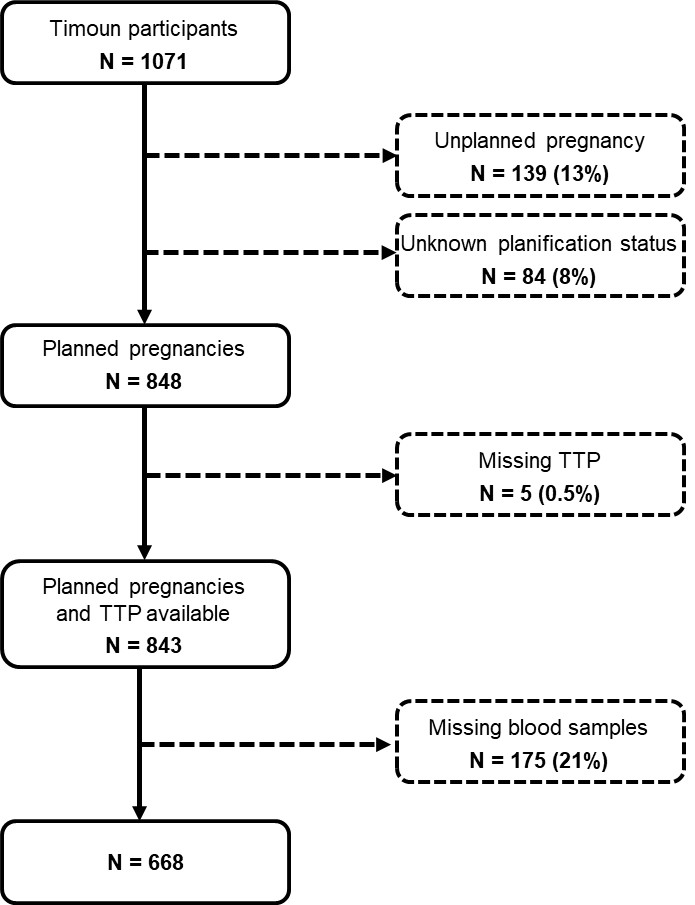

Supplement: Supplementary file 3 — Supplementary Material 3. [file 12940_2025_1233_MOESM3_ESM.jpg]

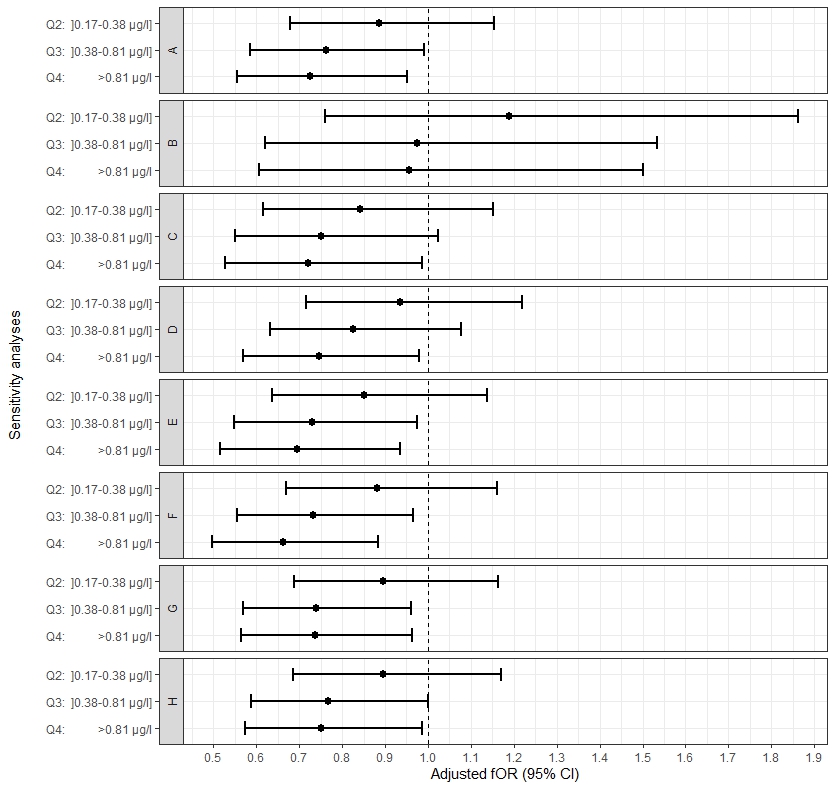

Supplement: Supplementary file 4 — Supplementary Material 4. [file 12940_2025_1233_MOESM4_ESM.jpeg]
