# Supplementary material for: Chlordecone exposure in women and time to pregnancy: the Timoun cohort study in Guadeloupe, French West Indies
Source: Environ Health. 2025 Oct 16;24:78. doi: 10.1186/s12940-025-01233-z (PMC12529803; doi:10.1186/s12940-025-01233-z)
Supplement: Supplementary file 2 — Supplementary Material 2. [file 12940_2025_1233_MOESM2_ESM.docx]

**Figure 1**. Sensitivity analysis. Points represent adjusted fOR and lines represent their 95% CIs for the association between chlordecone concentration and TTP in various sensitivity analyses. Models: A) main analysis (*n*=668); B) analysis restricted to primiparous women (*n*=243); C) analysis excluding women who conceived in the first month after the cessation of contraception with TTP=1 (*n*=510) ; D) analysis excluding women using fertility treatment (*n*=650); Main analysis with censoring at: E) 7 months, F) 10 months and G) 15 months (*n=*668) ; H) adjustment for covariate determinants of TTP (origin, education and marital status) (*n*=668).

**Fig. A.1** Flowchart of the study sample. Timoun mother-child cohort study, Guadeloupe, French West Indies, 2004-2007.

**Fig. A.2** DAG for chlordecone and time to pregnancy
